# Decoding the dynamic representation of musical pitch from human brain activity

**DOI:** 10.1038/s41598-018-19222-3

**Published:** 2018-01-16

**Authors:** N. Sankaran, W. F. Thompson, S. Carlile, T. A. Carlson

**Affiliations:** 1 School of Medical Sciences, University of Sydney, Sydney, NSW Australia; 2 ARC Centre of Excellence in Cognition and its Disorders, Macquarie University, Sydney, NSW Australia; 3 Department of Psychology, Macquarie University, Sydney, NSW Australia; 4 School of Psychology, University of Sydney, Sydney, NSW Australia

## Abstract

In music, the perception of pitch is governed largely by its tonal function given the preceding harmonic structure of the music. While behavioral research has advanced our understanding of the perceptual representation of musical pitch, relatively little is known about its representational structure in the brain. Using Magnetoencephalography (MEG), we recorded evoked neural responses to different tones presented within a tonal context. Multivariate Pattern Analysis (MVPA) was applied to “decode” the stimulus that listeners heard based on the underlying neural activity. We then characterized the structure of the brain’s representation using decoding accuracy as a proxy for representational distance, and compared this structure to several well established perceptual and acoustic models. The observed neural representation was best accounted for by a model based on the *Standard Tonal Hierarchy*, whereby differences in the neural encoding of musical pitches correspond to their differences in perceived stability. By confirming that perceptual differences honor those in the underlying neuronal population coding, our results provide a crucial link in understanding the cognitive foundations of musical pitch across psychological and neural domains.

## Introduction

Context is critical to perception. In music, two physically identical tones heard in different contexts may bear little resemblance. This distinction arises because the *tonality* or *key* of the musical context assigns a *unique function* to each pitch^1^. The two tones, despite being acoustically identical, differ in their tonal function and are therefore perceptually distinct.

More generally, tonality describes the tendency for pitch relationships to be oriented around a central pitch termed the *tonic*^2^. This organization establishes a hierarchy of perceived stability amongst the pitch-classes. The tonic occupies the most stable position, and other classes vary in perceived stability depending on their harmonic relationship to the tonic. Krumhansl (1979)^3^ investigated the mental representation of musical pitch by measuring the perceived similarity between different pitch-classes. From this work, a geometric model was derived that places each pitch-class on the surface of a cone. By making explicit the perceived relatedness between pitch-classes, the conical model forms a cornerstone in our understanding of the cognitive processing of musical pitch. The model predicts that *in-key* and *out-of-key* classes are distinct from one another, occupying two distant regions of the representational space. The in-key classes are situated near the apex of the cone and are thus perceptually similar to one another. Conversely, the out-of-key classes are dispersed around the basal end of the cone, and are therefore perceptually distant from one another, and from the in-key classes.

In the neural domain, studies have identified features of pitch-evoked cortical responses^4–11^, and described the anatomical and functional brain regions implicated in the neural processing of tonal structure^12–17^. Recording electroencephalographic (EEG) activity during melodic listening, Brattico *et al*.^4^ found that out-of-key pitches elicited an early pre-attentive cortical negativity, suggesting that tonal properties of musical pitch are automatically processed in the cortex. Recording EEG from trained musicians, Krohn *et al*.^5^ found that the amplitude of pitch-evoked response components were modulated based on the perceptual stability of the evoking pitch-class, suggesting a stored representation of hierarchical pitch structure in cortex. While these studies flag the presence of tonal-schematic processing, relatively little is known about the explicit representational structure of musical pitch in the cortex.

To examine the relatedness of different musical keys in the brain, changes in fMRI activity have been measured as a musical passage modulates across multiple tonal regions^18^. While the relatively slow fMRI responses are sensitive to the gradual accumulation of pitch-distributional information across a musical passage, evidence suggests that the cognitive basis of tonality arises from the perception of individual pitches given a tonal context^19^. We therefore measured highly temporally-resolved responses to individual tones presented within a musical context. By examining distinctions in the brain’s response to various pitches of differing tonal function, the current study provided a neural analogue to prior psychological models and evaluated their specific predictions: how distinctly does the brain represent each pitch-class relative to one another, and well do these neural distinctions align with perceptual differences between musical pitches?

**Figure 1:**
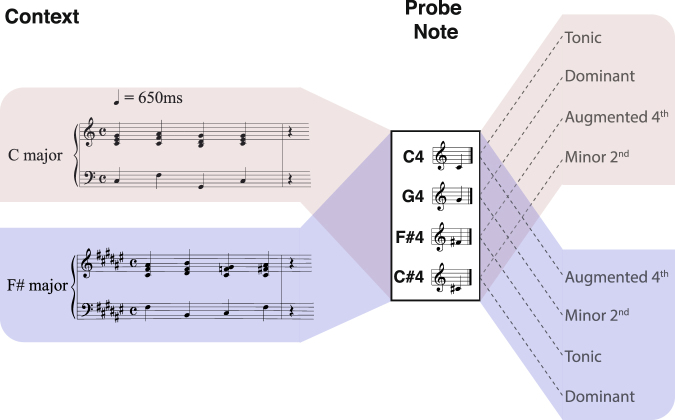
Experimental Paradigm. Each trial consisted of a four-chord tonal context followed by a single probe-tone. Stimuli were piano tones. Each chord and tone was 650 ms in duration and a silent interval of 650 ms separated the last chord and probe-tone. Contexts were either in the key of C major (top) or F# major (bottom). Subsequent probe-tones were C4, G4, F#4 or C#4. When the context was in the key of C major, the former two probe-tones were “in-key” (tonic & dominant), while the latter two probe-tones were “out-of-key” (augmented 4^th^ & minor 2^nd^). When the context was in the key of F# major, this mapping reversed.

To answer these questions, we focused on a set of four pitch-classes whose harmonic and perceptual properties make them strong candidates for observing a clear representational structure in the brain^20,21^. Two pitch-classes (the *tonic* and *dominant*) were highly stable scale notes within the prevailing key, while the other two pitch-classes (the *minor 2*^*nd*^ and *augmented* 4^th^) were out-of-key and highly unstable. Each tone was presented within a tonal context to trained musicians (Fig. 1). Magnetoencephalography (MEG) was used to measure the neural response patterns evoked by each class. We then used Multivariate Pattern Analysis (MVPA) to “decode” the pitch that listeners heard based on the underlying neural activity. Using decoding accuracy as a proxy for representational distance, we characterized the structure of the neural representation of musical pitch and compared this structure to several perceptual and acoustic candidate models. The observed neural representation was best accounted for by a model derived from the *Standard Tonal Hierarchy*^20^, indicating that differences in the neural encoding of musical pitch correspond to differences in their perceived stability.

## Results

### Decoding pitch-class from neural activity

Our goal was to map a set of pitches to points in representational space based on the similarity of their neuronal population activity. To achieve this, we trained a machine-learning classifier to measure the neural discriminability between all pairwise combination of tones at each time-point in the MEG data. The accuracy with which the classifier could discriminate between neural responses for a given pair of tones provided a measure of their neural representational distance; and the complete set of pairwise distances defined the geometry of the stimulus’ representational structure in the brain^22,23^. This approach affords greater power over straightforward activation-based measures (such as ERPs) because it preserves the rich dimensionality of the measured cortical response patterns, allowing a more fine-grained characterization of neural activity. Moreover, the MEG data was sampled at 200 Hz (see methods), and by applying the analysis to each time point, we were able to track the emergence of the brain’s representational structure across time.

**Figure 2:**
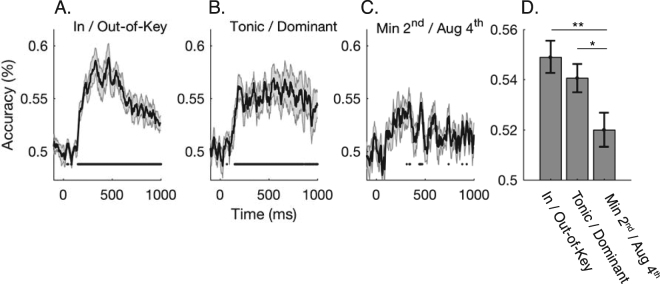
Decoding pitch-class from MEG activity. Neural distinctions were probed at each time-point from −100 ms to 1000 ms relative to onset of each probe-tone. Performance is averaged across both tonal contexts (C major and F# major) and all subjects. Statistically significant time points are indicated by the black points underneath each curve (p < 0.01; Wilcoxon sign-rank test, corrected by controlling the false discovery rate). Shaded regions indicate standard errors. (**A**) Classification accuracy for discriminating between in-key and out-of-key tones. (**B**) Accuracy for decoding the two in-key tones (tonic/dominant). (**C**) Accuracy for decoding the two out-of-key tones (minor 2^nd^/augmented 4^th^). (**D**) Time-averaged decoding performance for each of the distinctions assessed in (**A**–**C**) over 250–600 ms (the period of maximal context-related effects; see section 2.4). Significance is indicated by asterisks where *p < 0.05; **p < 0.01 (Bonferroni corrected Wilcoxon sign-rank test).

Perceptually, in-key pitches sound stable with respect to the tonal context, while out-of-key pitches sound unstable^19,21^. Is this psychological distinction evident neurally? To answer this question, we sought to discriminate between responses corresponding to in-key tones from that of out-of-key tones. Figure 2A displays the time-varying decoding performance averaged across subjects, tonal contexts, and pairwise combinations of in-key/out-of-key tones. As expected, classification performance at onset (*t* = 0) was at chance (50%) because stimulus-related information was yet to activate the cortex. However, by 150 ms we could successfully discriminate between the neural activity corresponding to in-key and out-of-key pitches, with maxima in classification accuracy occurring at 350 ms.

We next considered the brain’s distinction between the two in-key tones by evaluating the classifiers ability to discriminate between evoked responses corresponding to the tonic and dominant. To this end, we significantly decoded the tonic-dominant distinction from 150 ms onwards (Fig. 2B). In this case however, the peak accuracy was lower than when discriminating between in-key/out-of-key tones, suggesting that the in-key tones are more similar to each other than they are to out-of-key tones. Lastly, we attempted to decode activity corresponding to the two out-of-key tones (augmented 4^th^/minor 2^nd^). Here, decoding performance failed to rise above chance classification for any sustained period (Fig. 2C), suggesting that the neural distinction between the out-of-key tones is relatively weak. Indeed, when examining the time-averaged decoding performance (Fig. 2D), we found that the neural distinction between the out-of-key tones was significantly weaker than both the in-key/out-of-key (Z = 2.97, p = 0.006) and the tonic/dominant (Z = 2.41, p = 0.03) distinctions.

### Characterizing the neural representational structure of pitch-class

**Figure 3:**
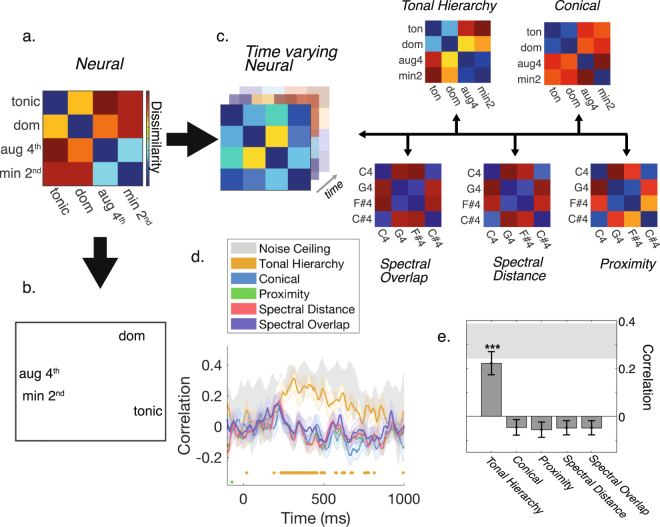
Representational similarity analysis of pitch-class. (**a**) Neural dissimilarities summarized in a time-averaged Representational Dissimilarity Matrix (RDM). (**b**) Multidimensional scaling (MDS) applied to the time-averaged neural RDM provides an intuitive visualization of the representational structure of musical pitch in the brain. (**c**) The time-varying neural structure is indexed with a new RDM at each time-point and compared with three candidate models. (**d**) Time-varying correlation (Kendall’s Tau_A_ rank-order) between the observed neural structure and each of the candidate models. Significance is indicated by the points below the curves (p < 0.05; randomization test; FDR corrected). Shaded regions indicate standard errors. (**e**) Time-averaging the neural-to-candidate correlations over 250–600 ms reveals that neural dissimilarities are significantly correlated with the Tonal Hierarchy (p < 0.05; randomization test), with the average correlation closely tracking the noise-ceiling (indicated by the shaded region). Bars indicate standard errors.

We next examined the brain’s collective representation for all four pitch-classes. The decoding performance for all pairwise combinations of tones defines a multidimensional geometrical structure in neural representational space, which can be summarized in a *Representational Dissimilarity Matrix* (RDM)^22,24^. The four different pitches are indexed along the rows and columns of the RDM, with each cell of the matrix indicating the measured neural dissimilarity. Figure 3a shows the RDM for the data time-averaged across 250–600 ms, a period chosen based on the period of maximal context-related information in the brain (see section 2.4).

We used multidimensional scaling (MDS) to visualize the structure of the RDM in two dimensions (Fig. 3b; see methods for details). In this arrangement, the distances between different pitch-classes directly relate to the measured neural dissimilarities, providing an intuitive and data-driven illustration of the representational structure. Most evident within this geometry is the brain’s distinction between the in-key and out-of-key tones, indicated by their locations to the right and left of the structure respectively. The geometry of the MDS solution also makes evident the neural distinction between the two in-key tones (tonic and dominant), while the two out-of-key classes are more similarly represented in the brain.

### Comparing neural dissimilarities with perceptual and acoustic models

We next tested several candidate models that might explain the currently observed neural representation. Two models were derived from behavioral data; one based on inter-class Euclidean distances in the *Conical* model, thus reflecting the perceived similarity between pitch-classes^2^ and another derived from the *Standard Tonal Hierarchy*^21^, where dissimilarities correspond to the difference in perceived tonal stability between all pairs of pitches. To assess the extent to which sensory differences might account for current neural dissimilarities, we tested a model of *Spectral Distance*. Specifically, spectrograms for each probe-tone were first extracted using a biologically inspired model of the auditory periphery^25^. We then computed the Euclidean distance between each pairwise combination of spectrograms. Next, we tested a candidate model based on the *Spectral Overlap* between the tonal context and each probe-tone. Although the context and probe-tones were separated by a 650 ms silent period in the current study, models of auditory short-term memory involve time constants of up to 4 seconds^26,27^. Thus, the neural distinctions between probe-tones may have been driven by the sensory memory of the context. Spectral overlap was computed by finding the reciprocal of the Euclidean distance between spectrograms corresponding to the context and each probe-tone. Dissimilarities were then determined by calculating the difference in spectral overlap for each combination of context and probe-tone, averaged across the two tonal contexts. Finally, we evaluated the hypothesis that listeners may simply be representing each probe-tone in terms of its pitch-value alone (i.e. fundamental frequency). We therefore tested a model - termed *Proximity -* that was based solely on the differences in semi-tone pitch-height between each pair of probe-tones. Each model makes explicit predictions about the structure of the brain’s representation of pitch-class and can be expressed as a candidate RDM (Fig. 3c).

To evaluate each model’s capacity to predict the observed neural representation, we constructed a set of time-varying RDMs from the MEG decoding accuracies and compared this time-evolving representation to each model’s prediction using *Representational Similarity Analysis*^22,28,29^. Figure 3d shows the time varying correlation between each candidate RDM and the time varying neural RDMs, averaged across both tonal contexts. We found that neural dissimilarities were significantly correlated with the Tonal Hierarchy model during a period starting around 250 ms, suggesting that the brain’s representation of the stimuli is best explained by differences in perceived stability between the four classes. Conversely, the all other RDMs provided little to no explanatory power. Tonal Hierarchy model correlations closely tracked the noise ceiling^24^, indicating that the model provided a good fit despite inherent noise in the MEG data. Finally, to summarize the modelling, we computed average correlations over the putative period of maximal context-processing (250–600 ms; see section 2.4). Again, the Tonal Hierarchy model was the best fit for the data (Fig. 3e); the average correlation is both significant and close to the noise ceiling.

### Dissociating tonal schema from acoustics

In addition to modelling, we wished to verify that the currently observed representation of pitch-class was based upon the neural processing of tonal-schema rather than afferent acoustic information. Our experimental paradigm deliberately included two different tonal contexts to re-map the harmonic function of each given probe-tone (see Fig. 1). Specifically, tones that were *in-key* in C major were *out-of-key* in F# major (and vice-versa). This enabled us to examine the brain’s distinction between two acoustically identical tones that were preceded by different contexts (thus differing solely in their pitch-class). Decoding ‘across-context’ in this fashion allowed us to examine neural distinctions between stimuli with identical sensory features, thereby isolating the effects of tonality.

**Figure 4:**
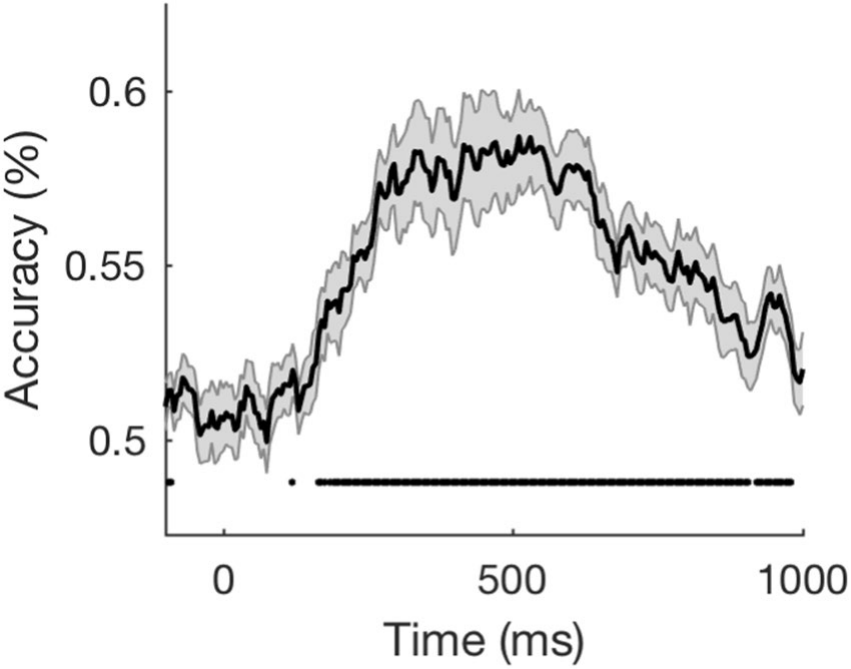
Decoding acoustically identical probe-tones. Applying MVPA to decode the pitch-class from physically identical tones that were preceded by different tonal contexts.

The results, averaged across the four probe-tones, are shown in Fig. 4. The tonal function could clearly be decoded from the neural response patterns of two acoustically identical tones. This distinction in the brain first emerged at 160 ms, with performance peaking at 58% correct for a sustained period from 250–600 ms. Crucially, performance was similar or better than when decoding pitch in the presence of acoustical differences (section 2.1). This result, in addition to the failure of spectral distance and proximity models to account for neural dissimilarities (Fig. 3d,e), suggests that the representation of pitch-class (Fig. 3a) reflects primarily the processing of tonal schema in the brain.

Note that stimuli in Fig. 4 were preceded by acoustically different contexts. As a result, there are indications of significant decoding before the onset of the probe-tone (i.e. at time-points less than zero). Crucially however, decoding accuracy is around chance at time zero. This result, in addition to the failure of the spectral overlap model to explain neural distinctions (Fig. 3d,e), suggests that the silent period separating context and probe-tone (650 ms) was sufficiently long enough to ensure that the sensory processing of the context did not influence the subsequent evoked response to probe-tone. As such, we believe that current neural distinctions between pitch-classes were driven by the processing of probe-tones within the tonal framework imparted by the context.

## Discussion

We characterized the brain’s representation for a set of four pitches that differed in their tonal function by examining differences in their evoked MEG response patterns using multivariate pattern analysis (MVPA). Consistent with prior Event Related Potential (ERP) research examining the cortical processing of tonal structure, these neural distinctions first emerged from 150 ms and were maximal from approximately 250–600 ms^7,30^. We uncovered a neural representation that placed in-key and out-of-key classes in distant regions of representational space. The in-key classes were also distant from one another. Conversely, the out-of-key classes were closely related. Because the collective representation was based on *dissimilarities* between classes, we could directly compare the observed neural structure with prior behavioral and acoustic models without the need for a direct correspondence mapping (Fig. 3c). Our principal finding was that neural dissimilarities were correlated with differences in the *Standard Tonal Hierarchy*^19,21^, suggesting that the difference in perceived stability between two pitch-classes can be conceptualized in terms of representational distance in the brain.

At a fundamental level, a common finding across behavioral and neural domains is that the representational distance between two tones forming an acoustically fixed interval varies depending on their respective classes within the tonal context. However, the nature of this variation is corroborated somewhat differently. The conical model^3^ suggests that in-key tones are proximate and out-of-key tones are distant from one another, whereas the current results suggest the opposite. Though seemingly contradictory, the two findings may not be mutually exclusive. The current method models the ‘distinctiveness’ of pitch-classes based on the dissimilarity of their evoked neural features. Because the dominant has a distinctive harmonic function relative to the tonic, it is likely that trained listeners possess a schema with which to separate the two pitches. In contrast, the augmented 4^th^ and minor 2^nd^ have no such functional harmonic relationship. It seems, therefore, that in the absence of a clear schema, the brain’s representation of the two out-of-key pitches converges. In the conical model however, *distance* reflects the degree to which two tones are perceived to be musically associated. In such a framework, the tonic and dominant may indeed be related. Both classes have a high probability of joint occurrence within tonal passages or as constituent pitches of chords^31^. Conversely, out-of-key classes may be considered musically unrelated; the occurrence of an out-of-key tone is likely to be followed by a resolution to the nearest in-key (rather than another out-of-key) pitch-class. By distinguishing between these two concepts – the *distinctiveness* of two pitches as opposed to how well they musically *fit* – the current study helps to clarify the complex nature of representational distance.

It should also be noted that the current investigation presented single tones following a tonal context, whereas the conical model arose from similarity ratings of tone *pairs* presented within a tonal context. Nonetheless, the conical model provides a general account of the psychological similarity of pitch-classes. Therefore, it is valuable to assess the extent to which this model can be generalized beyond the specific behavioral methodology from which it arises. As such, we tested the hypothesis that perceptually dissimilar tones (as described by the conical model) also have dissimilar patterns of brain activation when presented in isolation.

In order to further understand the neural basis of tonality, future work should extend the stimulus set to include all twelve pitch-classes so that more precise modelling may begin to establish the optimal combination of perceptual, sensory and acoustic features that explain the brain’s response to tonal pitch. For example, it may be the case that when all pitch-classes are considered, a mixed model of sensory and schematic features may be needed to account for neural distinctions.

We assessed the ability of classifiers to discriminate between MEG responses evoked by tones differing in their tonal functions. Using classification accuracy as a measure of representational distance, we characterized a representation for a set of musical pitches and showed that their collective representational structure correlates with the respective differences in their perceived tonal stability. Our results provide a crucial link between musical pitch perception and the underlying neural activity from which it materializes. Music psychology has long held that the cognitive basis of tonality can be derived from the “Tonal Hierarchy” – the pattern of stability across different pitches within a musical context. The current results strengthen this notion by showing consistency in the relations between tones across neural and perceptual domains.

## Materials and Methods

### Experimental Design

Thirteen trained musicians with a minimum of 5 years of formal training (Mean = 11.8 years, SD = 3.0) were recruited from the Sydney Conservatorium of Music, the Australian Institute of Music and Macquarie University. The sample size was not pre-determined, but rather testing was terminated once trends in the decoding analyses displayed sufficient statistical power (see statistical analysis below). All subjects reported having no known hearing loss or brain abnormalities, and did not possess absolute pitch. The study was approved beforehand by the Human Research Ethics Committee at Macquarie University (REF 5201300804) and all methods were carried out in accordance with the stated guidelines. Informed consent was obtained prior to testing, after all experimental details and potential risks were explained.

Each trial consisted of a tonal context followed by a probe-tone. Contexts were either in the key of C major or F# major, and consisted of four major chords written in four-part harmony outlining an I-IV-V-I harmonic progression. Subsequent probe-tones were either C4 (261.6 Hz), G4 (392.0 Hz), F#4 (370.0 Hz) or C#4 (277.2 Hz). Within each key, two versions of the tonal context were presented: one in which chords contained tones that were also probe-tones (for example, in a C major context the C4 and G4 were both physically present in the preceding chords), and an alternate version in which these constituent tones were transposed an octave above or below their original position in the chords (i.e. a chord inversion). The inclusion of this alternate tonal context enabled us to assess the effect, if any, of the acoustic spectral overlap between context and probe. We found no significant difference in classification performance when the data were divided into the ‘f0-overlap’ and ‘no-f0-overlap’ conditions (Wilcoxon sign rank test, False Discovery Rate corrected p > 0.05). All reported results are therefore based on an analysis of trials grouped across the two versions of the tonal context.

Stimuli were piano tones recorded at 44.1 kHz. Tones were sampled in Max/MSP (Cycling’74, San Francisco, CA) to form chords and probe-tones that were 500 ms in duration with an additional 150 ms decay. Following the last chord of the context, a silent period equivalent to one beat in the tempo of the passage (650 ms; roughly 92 beats per minute) was inserted. This temporal separation was intended to prevent the sensory processing of the context from influencing the evoked response to probe-tones (see Fig. 4), while also maintaining metric regularity. Prior to testing, all probe-tones were passed through a time varying loudness model^32^ to normalize for differences in perceived loudness. For each tone, the *maximum short-term-loudness* (STL_max_) was computed and normalized to the mean value of all four tones. Differences in STL_max_ between the four probe-tones did not exceed 3 phones.

Each participant’s MEG data were collected in a single hour-long session. The session was sub-divided into 8 testing blocks separated by one-minute breaks, during which subjects watched and listened to a movie. Each block consisted of 80 trials in a single tonal context (C major or F# major), with adjacent blocks alternating between the two keys. The two versions of each tonal context (with and without shared probe-tone pitches) were presented in randomized fashion with equal probability within a block. ERP studies indicate that increases in the probability of syntactically irregular trials results in decreased effect sizes^7^. We therefore opted for an in-key to out-of-key presentation ratio of 6:4, resulting in a total of 192 in-key and 128 out-of-key observations within each tonal context. After each trial, participants responded as to whether the probe-tone was ‘in-key’ or ‘out-of-key’, registering their response by pressing one of two buttons. This was done to ensure participants were attending to the stimuli^33^. Participants used their left and right thumbs to register the two different responses. The assignment of in-key/out-of-key to left/right button was interchanged every two blocks to control for the effect, if any, of motor activity on the measured neural responses. Once the response was registered, inter-trial-intervals were randomly jittered between 0.5–1 seconds. Before testing, subjects completed a training session consisting of 20 trials with an identical behavioral task to that of the MEG recording session. Feedback was provided after each training trial and the experimenter ensured that subjects could perform the task (using a threshold of ≥90% correct) before proceeding to the MEG recording session. No trial-by-trial feedback was provided during the MEG recording; however, subjects were informed of their accuracy after each block. On average, subjects responded correctly on 96% of the trials (SD = 5.9%).

### Apparatus

Data were collected with a whole-head MEG system (Model PQ1160R-N2; KIT, Kanazawa, Japan) consisting of 160 coaxial first-order gradiometers with a 50 mm baseline^34,35^. Prior to recording, five marker coils were placed on the participant’s head, their positions were registered and the participant’s head shape were measured with a pen digitizer (Polhemus Fastrack, Colchester, VT, USA). MEG data was bandpass filtered online from 0.1–200 Hz using first-order RC filters and digitized at 1000 Hz. Participants were in a supine position in the scanner and were instructed to direct their gaze at a fixation cross. Both the fixation cross and experimental instructions were projected by an InFocus IN5108 LCD back projection system (InFocus, Portland, Oregon, USA) to a screen located above the participant at a viewing distance of 113 cm. Sound stimuli were delivered via Etymonic ER-30 insert headphones at a sampling frequency of 44.1 kHz.

### MEG Pre-processing

All pre-processing was performed in MATLAB. Data was epoched from 0.1 s before to 1 s after onset of probe-tones. To improve the signal to noise ratio while still retaining temporal resolution, the MEG data was downsampled to 200 Hz with a low-pass Chebyshev Type 1 filter. Applying Principal Components Analysis (PCA) has been found to be an efficient pre-processing step for optimizing (or near- optimizing) data for MEG decoding analyses. In a single step PCA reduces the dimensionality of the data, and obviates the need for additional artefact rejection or de-noising procedures, as the classifiers can “learn” to suppress nuisance variables isolated by PCA, e.g. eye-blinks and environmental noise^36^. In the present study, PCA was applied to each participant’s dataset of [640 trials × 160 channels × 1100 ms] and the first n components accounting for 99% of the variance for each subject were retained for the decoding analysis. On average, PCA reduced the dimensionality of the dataset from 160 sensor channels to 26 principle components.

### Time-series Decoding Analysis

Multivariate pattern analysis (MVPA) of MEG data was performed in MATLAB. For each set of pre-processed data, we used a naïve Bayes implementation of linear discriminate analysis (LDA)^37^ to perform single-trial classification for each pairwise combination of pitch-classes at each time-point. Generalization of the classifier was evaluate using k-fold cross validation with a 9:1 training to test ratio. In this procedure, the MEG data for all trials corresponding to the two classes being decoded were randomly assigned into 10 bins of equal size, with matched numbers of observations across the two classes in each bin. Nine of the bins were pooled to train the classifier, and the trials in the remaining bin were used to test the classifier. This procedure was repeated 10 times such that each trial was included in the test bin exactly once. Decoding was performed with a sliding time-window to assess the time-varying ability of classifiers to discriminate between neural activity corresponding to two given pitch-classes. To provide more observations in each classification run, we used a window size of 25 ms and a step-size of 5 ms. This meant that each classification run was based on data from the 5 most recent points in the timeseries. Because of the temporal ‘smearing’ associated with such windowing, the reported onset times for significant decoding are conservative estimates.

### Representational Similarity Analysis (RSA)

Decoding every pairwise combination of the four pitch-classes occurring within the two tonal contexts resulted in an 8 × 8 Representational Dissimilarity Matrix (RDM) for each subject at each time point. From this, we created a single 4 × 4 RDM (Fig. 3a) that was averaged across subjects, the two tonal contexts, and time-points from 250–600 ms (the period of maximal context decoding - see Fig. 4). Multidimensional Scaling (MDS; Kruskal’s normalized stress1 criterion) was used to illustrate the structure of the RDM in two dimensions. MDS aims to spatially represent the RDM whilst preserving the original distances as much as possible. The loss function or *stress* of the solution indicates how faithfully MDS preserves the distances. Typically, the stress is minimized with higher-dimensional solutions. In our case however, the 2-D solution produced negligible stress (stress = 8.5819 × 10^−7^), indicating that the dimensionality was sufficient to visually depict the RDM. We constructed five model RDMs based on perceptual and acoustic properties that may account for the neural dissimilarities observed. The *Conical* RDM was based on the Euclidean distances between pitch-classes in Krumhansl^3^. The *Tonal Hierarchy* RDM was based on the difference in stability ratings reported in Krumhansl & Kessler^21^. Stimulus spectrograms were computed by passing the raw audio through a model of the auditory periphery^25^. The model consisted of three main stages: (1) a cochlear filter bank comprised of 128 asymmetric filters uniformly distributed along a logarithmic frequency axis (2), a hair cell stage consisting of a low-pass filter and a nonlinear compression function, and (3) a lateral inhibitory network approximated by a first-order derivative along the tonotopic axis followed by a half-wave rectifier. We constructed an RDM of *Spectral Distance* between probe-tones by calculating the Euclidean distance between the respective spectrograms across all 128 frequency bands. For each context, a *Spectral Overlap* RDM was computed by calculating the differences in the Euclidean distance between the context spectrogram and each of the probe-tone spectrograms. The resultant RDM had similar patterns of dissimilarity for both tonal contexts (C major and F# major). For this reason, we averaged the Spectral Overlap RDM across both contexts for further analysis. Finally, the *Proximity* RDM was based on the semitone difference in pitch-height between probe-tones. Using the RSA framework^22,24^, we studied the brain’s emerging representation by comparing each model RDM with our empirical time-varying MEG RDM.

### Statistical Analysis

All statistical analyses were performed on all subjects (N = 13). Decoding performance is reported in terms of balanced accuracy (the mean of percent-correct in class A and percent-correct in class B). Time-series and time-averaged decoding performance was tested for significance using a two-sided Wilcoxon signed rank test. To correct for multiple comparisons, we controlled the false discovery rate (FDR)^38,39^ with α = 0.05. Correspondence between neural and model RDMs was assessed by computing Kendall’s Tau_A_ (i.e., a rank-order correlation)^24^ for each time point and each subject, producing a time-varying correlation between each model and MEG data. Significance of model correlations at each time-point was assessed using randomization testing. Briefly, the class labels on RDMs being compared were randomly re-assigned before computing correlation, and this process was repeated 10,000 times to define the null distribution at each time-point. Significant time-points were identified as those whose correlation values lay outside the 95% confidence intervals of the null distribution. Multiple comparisons were accounted for by controlling the false discovery rate (α = 0.05). We used the ‘noise ceiling’ as a benchmark for testing model performance^24^. The noise ceiling estimates the magnitude of the expected correlation between the “true” model RDM and the empirical RDM given the noise inherent in the measurement.

### Data Availability

The datasets generated during and/or analysed during the current study are available from the corresponding author on reasonable request.
